# Multi-phase, multi-ethnic GWAS uncovers putative loci in predisposition to elite sprint and power performance, health and disease

**DOI:** 10.5114/biolsport.2025.147015

**Published:** 2025-02-04

**Authors:** Guan Wang, Noriyuki Fuku, Eri Miyamoto-Mikami, Masashi Tanaka, Motohiko Miyachi, Haruka Murakami, Braxton D. Mitchell, Errol Morrison, Ildus I. Ahmetov, Edward V. Generozov, Maxim L. Filipenko, Andrei A. Gilep, Valentina Gineviciene, Colin N. Moran, Tomas Venckunas, Pawel Cieszczyk, Wim Derave, Ioannis Papadimitriou, Fleur C. Garton, Sandosh Padmanabhan, Yannis P. Pitsiladis

**Affiliations:** 1 School of Sport and Health Sciences, University of Brighton, Eastbourne BN20 7SN, United Kingdom; 2 Graduate School of Health and Sports Science, Juntendo University, Chiba 270-1695, Japan; 3 Graduate School of Medicine, Juntendo University, Tokyo 113-8421, Japan; 4 Faculty of Sport Sciences, Waseda University, Saitama 359-1192, Japan; 5 College of Sport and Health Science, Ritsumeikan University, Shiga 525-8577, Japan; 6 School of Medicine, University of Maryland, Baltimore 21201, MD, United States; 7 Diabetes Association of Jamaica, Kingston 5, Jamaica; 8 Research Institute for Sport and Exercise Sciences, Liverpool John Moores University, Liverpool, United Kingdom; 9 Laboratory of Genetics of Aging and Longevity, Kazan State Medical University, Kazan, Russian Federation; 10 Department of Physical Education, Plekhanov Russian University of Economics, Moscow, Russian Federation; 11 Department of Molecular Biology and Genetics, Lopukhin Federal Research and Clinical Center of Physical-Chemical Medicine of Federal Medical Biological Agency, Moscow, Russian Federation; 12 Laboratory of Pharmacogenomics, Institute of Chemical Biology and Fundamental Medicine, Siberian Branch of the Russian Academy of Sciences, Novosibirsk, Russian Federation; 13 Novosibirsk State University, Novosibirsk, Russian Federation; 14 Laboratory of Molecular Diagnostics and Biotechnology, Institute of Bioorganic Chemistry of the National Academy of Sciences of Belarus, Minsk, Belarus; 15 Laboratory of Intermolecular Interactions, Institute of Biomedical Chemistry (IBMC), Moscow, Russian Federation; 16 Translational health research Institute, Faculty of Medicine, Vilnius University, Vilnius LT-08406, Lithuania; 17 Physiology, Exercise and Nutrition Research Group, Faculty of Health Sciences and Sport, University of Stirling, Stirling FK9 4LA, United Kingdom; 18 Lithuanian Sports University, Kaunas LT-44221, Lithuania; 19 Gdansk University of Physical Education and Sport, Gdansk 80-336, Poland; 20 Department of Movement and Sports Sciences, Ghent University, Ghent B-9000, Belgium; 21 Department of Physiology, Faculty of Science, Mahidol University, Bangkok 10400, Thailand; 22 Institute for Molecular Bioscience, University of Queensland, Brisbane 4072, QLD, Australia; 23 Institute of Cardiovascular and Medical Sciences, University of Glasgow, Glasgow G12 8TA, United Kingdom; 24 Department of Sport, Physical Education and Health, Hong Kong Baptist University, Kowloon Tong, Hong Kong; 25 Research Institute of Physical Culture and Sport, Volga Region State University of Physical Culture, Sport and Tourism, Kazan, Russian Federation; 26 Republican Scientific and Practical Center of Sports, Minsk, Belarus; 27 Meret Solutions Mental Health Association, Madison, United States

**Keywords:** Polypeptide N-Acetylgalacto-saminyltransferase 13, GWAS, Imputation, Meta-analysis, Genetic diversity, Functional annotations

## Abstract

The genetic underpinnings of elite sprint and power performance remain largely elusive. This study aimed to identify genetic variants associated with this complex trait as well as to understand their functional implications in elite sprint and power performance. We conducted a multi-phase genome-wide association study (GWAS) in world-class sprint and power athletes of West African and East Asian ancestry and their geographically matched controls. We carried out genotype imputation, replications for the top GWAS signal rs10196189 in two European cohorts, and gene-based and tissue-specific functional network analyses. For the first time, we uncovered the G-allele of rs10196189 in the Polypeptide N-Acetylgalactosaminyltransferase 13 (GALNT13) being significantly associated with elite sprint and power performance (P = 2.13E-09 across the three ancestral groups). Moreover, we found that GALNT13 expression level was positively associated with the relative area occupied by fast-twitch muscle fibers in the vastus lateralis muscle. In addition, significant and borderline associations were observed for BOP1, HSF1, STXBP2, GRM7, MPRIP, ZFYVE28, CERS4, and ADAMTS18 in cross-ancestry or ancestry-specific contexts, predominantly expressed in the nervous and hematopoietic systems. From the elite athlete cohorts, we further identified thirty-six previously uncharacterized genes linked to host defence, leukocyte migration, and cellular responses to interferon-gamma, and four genes – UQCRFS1, PTPN6, RALY and ZMYM4 – associated with aging, neurological conditions, and blood disorders. Taken together, these results provide new biological insights into the genetic basis of elite sprint and power performance and, importantly, offer valuable clues to the molecular mechanisms underlying elite athletic performance, health and disease.

## INTRODUCTION

Over the past three decades, extensive efforts have been made to uncover the genetic basis of elite athletic performance. In a series of updates of the gene map for human performance and fitness-related phenotypes between 2000 and 2015, over 200 autosomal genes and quantitative trait loci have been reported, often lacking replications [1, 2]. Despite persistent attempts to unveil the role of genes in human performance-related phenotypes [3–5], key genes and gene regulatory networks remain largely elusive. The underlying reasons for this lack of understanding are multi-faceted, such as the predominant reliance on a candidate gene approach, a primary focus on European populations, and small sample sizes that result in low statistical power to detect true effects. In recent years, the applications of genome-wide approaches [6], the shift to include genetically diverse populations [7, 8] and improved power [9] have propelled the field of genetic research to tackling complex phenotypes more effectively.

Here, we set out to conduct a multi-phase, cross-ancestry genome-wide association study (GWAS), comprised of elite sprint and power-oriented athletes of West African and East Asian ancestry. These athletes competed in major sprint, jump and throw events, representing the top-end of elite sprint and power performance by breaking world records or winning Olympic medals. We expect to uncover common genetic variants of moderate to large effect sizes on a sample size comparable to that of the landmark GWAS of age-related macular degeneration [10]. For the first time, these unique cohorts enabled us, with greater confidence, to explore both cross-ancestry and ancestry-specific genetic variations across the genome and their regulatory functions in association with elite sprint and power-oriented athletic status. In addition, we hypothesize that the genetic architecture underlying elite sprint and power performance would have significant implications for future research of human health and disease, particularly in musculoskeletal, metabolic, immunological and neurological conditions that share common biological pathways with this trait.

## MATERIALS AND METHODS

### Sample collection

*Elite Jamaican (Jam) and African-American (A-A) sprint cohorts (of West African ancestry)* [11, 12]*:* These cohorts comprised Jam and A-A sprint athletes of the highest caliber and geographically matched controls. One hundred and sixteen Jam athletes (60 male, 56 female) and 311 matched controls (156 male, 155 female) were recruited, as previously described [13]. Among them, 71 competed in the 100–200 m sprinting events, 35 in the 400 m, and 10 in the jumping and throwing events. They were classified as national-level athletes (n = 28) competed in Jamaica and the Caribbean and international-level athletes (n = 88) competed at major international competitions for Jamaica. Among the international-level athletes, 46 had won medals at the international events or held world records in sprinting.

One hundred and fourteen A-A sprint athletes (62 male, 52 female) and 191 matched controls (72 male, 119 female) were recruited, as previously described [13]. Among the athletes, 48 specialized in the 100–200 m sprinting events, 42 in the 400 m and 24 in the jumping and throwing events. The athletes included 28 competing at the national level and 86 at the international level, with 35 having won medals at international competitions or set sprint world records. An additional 350 A-A controls were incorporated from published dataset [14], to boost the number of A-A controls for this study.

*Elite Japanese (Jpn) sprint athlete cohort (of East Asian ancestry):* This cohort included 54 (48 male, 6 female) international-level sprint athletes from Japan, but they were not necessarily medallists. One hundred and eighteen geographically matched controls (38 male, 80 female) were recruited from the general population.

*Elite European athlete cohorts:* Cohort 1 comprised 133 sprinters, 234 power-oriented athletes, 451 endurance athletes and 1,525 controls from Belarus, Lithuania, and Russia. Cohort 2 consisted of 171 sprinters, 168 power-oriented athletes, 252 endurance athletes and 595 controls from Australia, Belgium, Greece, and Poland. All athletes were national-, and international-level, or were prize winners of these events.

*Muscle biopsy samples:* These samples were collected from 23 physically active Russian males as previously described [15] for muscle fiber composition and *GALNT13* gene expression analyses.

Ethics Committee of the University of West Indies, Jamaica (protocol number: ECP 121, 2006/2007), the Institutional Review Board of Florida State University, USA (D5.158), the Institutional Review Board of Juntendo University, Japan (SHSS 2022-137), the Institutional Review Board of Tokyo Metropolitan Institute of Gerontology, Japan (TMIG 19-3784), the Institutional Review Board of the National Institute of Health and Nutrition, Japan (KENEI 2-09), the Bioethics Commission of Institute of Bioorganic Chemistry of the National Academy of Sciences of the Republic of Belarus, Belarus (2017/03), the Lithuanian Bioethics Committee, Lithuania (69-99-111), the Ethics Committee of the Lopukhin Federal Research and Clinical Center of Physical-Chemical Medicine of the Federal Medical and Biological Agency of Russia, Russia (2017/04), the Institutional Review Board of the Children’s Hospital at Westmead, Australia (2003/086), the Royal Children’s Hospital Human Research Ethics Committee, Australia (35172), the Ethical Committee of Ghent University Hospital, Belgium (B67020097348), Aristotle University of Thessaloniki Research Committee, Greece (1895), and the Pomeranian Medical University Ethics Committee, Poland (BN-001/45/08) approved for this work. Written informed consent was obtained from all subjects. All research was performed in accordance with the relevant guidelines and regulations, and in accordance with the “Declaration of Helsinki”.

### DNA processing for GWAS

DNA was isolated from buccal cells or whole saliva. Participants were asked not to consume food or drink for at least 30 minutes before providing a sample. Buccal cells were collected by a trained individual by firmly rubbing a brush (Medical Packaging Corporation, Camarillo, CA, USA) against the inside of a participant’s cheek for at least 15 seconds. The head of the brush was then cut into a screw cap tube containing cell lysis solution (0.1 M Tris-HCl pH 8.0, 0.1 M EDTA; 1 % SDS). DNA was extracted using the QIAamp DNA Mini kit (QIAgen, Hilden, Germany) according to the manufacturer’s instructions with minor adjustments. Whole saliva was collected using the Oragene DNA Self Collection Kit (OG-250, DNA Genotek Inc., Canada). About 2 mL of saliva was collected and sufficiently mixed with the Oragene chemistry (DNA Genotek Inc., Canada) by repeated inversion for 10 seconds. DNA was extracted following the manual purification of DNA from 0.5 mL of sample using DNA Genotek’s prepIT∙L2P DNA extraction kit with minor adjustments. DNA was quantified using the Nanodrop Technologies Nanodrop ND-8000 Spectrophotometer (Wilmington, DE, USA).

Purified DNA samples from the Jam and A-A cohorts were shipped to Tokyo Metropolitan Institute of Gerontology, Japan, for genotyping on HumanOmniExpress (730,525 markers) and HumanOmni1-Quad Beadchips (1,134,514 markers) (Illumina, San Diego, California, USA). During transportation, DNA was stored in the sterile Thermo Scientific Matrix Storage Tubes (0.75 mL, 8 × 12 format; Thermo Fisher Scientific, Hudson, New Hampshire, USA) and was shipped with dry ice. DNA quality was re-evaluated using the PicoGreen Assay prior to the whole-genome genotyping; and only samples of at least 50 ng of DNA were taken forward.

### Discovery phase – part one: GWAS data analysis workflow

Illumina GenomeStudio Software (v2010.3), PLINK [16], and EIGENSTRAT [17] were used for converting array outputs to PLINK formats, performing genotype quality control (QC) and association testing, and conducting population stratification analyses, respectively.

Per-individual and per-marker QCs were initially performed in a cohort-specific manner. Single nucleotide polymorphisms (SNPs) met the following inclusion criteria: maximal proportion of missing SNPs per sample of 5% (267,969 Jam; 297,404 A-A; 48,490 Jpn; SNPs excluded); minor allele frequency (MAF) of 1% (9 A-A; SNPs excluded); minimum Hardy-Weinberg disequilibrium frequency *P*-value 1 × 10^−7^ (31 Jpn; SNPs excluded). Any samples failing per-sample QCs were removed, including discordant sex, low call rate below 95%, heterozygosity rate exceeding three standard deviations (SD) from mean heterozygosity, cryptic relatedness subject to proportion IBD (identity-by-descent) of 0.05 and visual inspection of the relationship between Z0 and Z1 values, and outliers of principal component analysis (Supplementary Table S1, and Supplementary Fig. S1 and S2). Genetic associations were evaluated using logistic regression for 22 autosomes in PLINK, assuming an additive effect in Jam and A-A and taking into account the top 10 principal components and genotyping center effect in the model, where appropriate. Standard allelic association analysis was performed in PLINK, comparing the allele-frequency differences in Jpn. Visualization of the GWAS results and regional associations was carried out in the Bioconductor package “karyoloteR” [18] and the LocusZoom [19], respectively; along with other packages, “Cairo” [20], “tidyr” [21], and “cpvSNP” [22], required for plotting.

### Discovery phase – part two: imputation and meta-analyses

Two imputation workflows were adopted: IMPUTE2 (phasing with SHAPEIT2 [23]) on the 1000G phase 3 reference panel [24, 25] and the Sanger Imputation Server on the African Genome Resources [26] (phasing with EAGLE2 [27], imputing with PBWT [28]) for each GWAS cohort. Post-imputation QC criteria included an imputation quality measure > 0.3 and a MAF ≥ 5%. Cohort-specific genetic associations were then tested using SNPTEST [29] under a frequentist additive model, conditioning on the top 10 principal components and genotyping centers, where appropriate. Meta-analyses using the inverse variance-weighted fixed-effect model of the three imputed GWAS cohorts were performed in METAL [30] subjected to genomic control (GC) correction.

### Replication phase: independent replications of rs10196189 in athletes of European descent

Genotyping of rs10196189 (*GALNT13*) was performed in two European cohorts (Cohort 1 and Cohort 2) comprising sprint and power-oriented athletes, endurance-oriented athletes, and their matched controls. Cohort 1 and 2 were independently organized and genotyped. Genetic associations were assessed using both additive and dominant genetic models within each replication cohort. Meta-analysis of the discovery and replication findings for rs10196189 was conducted in METAL [30], combining the association results from the Jam, A-A, and Jpn GWAS imputation cohorts, as well as Replication Cohort 1 “Other sprint/power oriented” and Replication Cohort 2 “Sprinters and Jumpers” sub-groups.

### Muscle fiber composition and GALNT13 gene expression analyses

Muscle fibre composition of musculus vastus lateralis was evaluated as previously described [31]. Briefly, vastus lateralis samples were obtained from the left leg using the modified Bergström needle procedure. Serial cross-sections (7 μm) were obtained from frozen samples using an ultratom (Leica Microsystems, Wetzlar, Germany). The sections were then incubated at room temperature in primary antibodies against slow or fast isoforms of the myosin heavy chains. Images were captured with a fluorescent microscope (Eclipse Ti-U, Nikon, Tokyo, Japan). The cross-sectional areas of fast- and slow-twitch muscle fibers were evaluated using the ImageJ software (NIH, USA). The subjects were restricted to training for one day, prior to the muscle biopsy of vastus lateralis of left leg in the morning.

Total RNA was isolated using the RNeasy Mini Fibrous Tissue Kit (Qiagen, Hilden, Germany) as previously described [15] and was treated with the Turbo DNA-free Kit (Thermo Fisher Scientific, Waltham, MA, USA) according to the manufacturer’s instructions. RNA-seq library was then prepared using the NEBNext Ultra II Directional RNA Library Prep Kit with the NEBNext rRNA Depletion Module (New England Biolabs, Ipswich, MA, USA) and was sequenced on a HiSeq system (Illumina, San Diego, CA, USA) for paired-end sequencing with a read length of 125 bp and an average read depth of 48.4 M [15]. Expression of the *GALNT13* gene was presented in transcripts per kilobase million for downstream analysis.

### Gene- and gene-set enrichment analyses

Gene-based analysis of the SNPTEST summary statistics was performed using MAGMA v1.09b [32] following Sanger imputation. SNPs were annotated to NCBI 37.3 and assigned to a gene if located within 5 kb up- or downstream of the gene region. The “multi” gene analysis model was applied, testing both “mean” and “top” SNP associations, which are sensitive to detect associations in high link-age disequilibrium (LD) regions of a gene using the sum of squared SNP *Z*-statistics as the test statistic or in the top proportion of SNPs using the lowest SNP *P*-value as the test statistic. Gene location files derived from NCBI 37.3 and LD estimations between SNPs using the African and East Asian reference populations of the 1000 Genomes Project were downloaded from the auxiliary files available at https://ctg.cncr.nl/software/magma. Fixed effects meta-analysis of the gene analysis results was performed in METAL across the Jam, A-A and Jpn cohorts, followed by competitive gene-set analysis using the MSigDB (v7.4) hallmark [33] (N = 50) and canonical pathways (C2:CP; N = 2,922) collections of functional gene sets. Multiple testing correction was applied according to the number of genes available for each analysis.

### LDAK gene-based heritability estimation

Gene-based heritability was estimated using individual-level SNPTEST results with restricted maximum likelihood (REML [34]) method, as implemented in LDAK5.2 [35]. Again, SNPs were annotated to NCBI 37.3 and assigned to a gene if located within 5 kb up- or downstream of the gene region. SNPs were scaled to the recommended power of -0.25, implying weak negative selection (See: dougspeed.com/gene-based-analysis/). Covariates were included to correct for population structure and genotyping center effect, where appropriate. Heritability estimates were provided on both the observed and liability scales by setting the population prevalence to 1% in the latter. This was based off a conservative estimate from the probabilities of competing in College Athletics provided by the US Federal associations, the National Federation of State High School Associations, and the National Collegiate Athletic Association (NCAA) [36] – the probabilities for women and men competing in Track and Field at the NCAA Division I level during 2018–19 were 2.8% and 1.9%, respectively. Estimates for competing in professional athletics or at the Olympic level in Track and Field are not available, but are presumed to apply to only a select few (well under 1%). Here we set an arbitrary liability scale to 1%, only as an indicator of low prevalence.

### Data-driven prediction for tissue-specific gene functions, regulation and GWAS re-prioritization in humanbase

To functionally annotate the findings from both SNP- and gene-based analyses, we utilized humanbase (https://humanbase.io/). Humanbase hosts a suite of machine-learning algorithms (Naïve Bayes, deep learning, and Supporter Vector Machine) to enable functional predictions of genomic variants and genes. By integrating 987 genomic datasets, consisting of ~38,000 conditions from ~14,000 publications, and by accessing their relevance in 144 tissues and cell lineages, it provides a high throughput and unbiased method to prioritize genes and genetic variations for follow-up. We applied tissue-specific functional network analysis (GIANT [37]), implemented in humanbase, for the top genes identified from the gene-based analyses including *GALNT13, STXBP2, BOP1, HSF1, GRM7, MPRIP, ZFYVE28* and *ADAMTS18*; the data plotted using cowplot [38]. In addition, we performed the Sei analysis [39] for six sentinel SNPs, representing independent regional signals resided near or in *GALNT13* (rs113303758, T/C), *STXBP2* (rs2303115, G/A), *BOP1* (rs4977199, T/G), *GRM7* (rs3864067, A/G), *MPRIP* (rs117143557, C/T), and *CERS4* (rs2927712, A/C), to investigate tissue- and cell-type-specific regulatory activities. We have also conducted functional module [40] detection in blood for the interferon-gamma response gene set, which includes 199 genes identified through gene-set enrichment analysis using MAGMA, to explore genetic associations with specific biological processes represented by cohesive gene clusters or functional modules, including potential associations involving functionally uncharacterized genes in the detected modules.

Lastly, we conducted NetWAS [37] analysis on nominally significant genes (*P* < 0.01) identified from the gene-based analysis, using a relevant tissue network representative of the top gene findings in each cohort. Specifically, 18,416 (Jam), 18,414 (A-A), and 18,317 (Jpn) and 13,301 (meta-analysis) genes were uploaded for gene reprioritization in the central nervous system, forebrain, brain, and caudate nucleus, respectively. The resulting positive NetWAS scores indicate genes that are more likely to be nominally significant. To further evaluate the reprioritized gene lists, we submitted them to Enrichr [41–43] for functional annotation, focusing on gene enrichment in “Diseases/Drugs” and “Cell Types” gene-set libraries.

### Statistical analysis

For the GWAS association results, a *P* value of 5E-05 was used as a suggestive cut-off. For the imputed datasets, results were filtered using *P* < 1E-05. The relationship between *GALNT13* gene expression and the relative area occupied by fast-twitch muscle fibers was analyzed using a Generalized Linear Model with a Gamma distribution and an “inverse” link function, adjusted for age, body mass index (BMI) and athletic levels, in R [44]. For the gene- and gene-set enrichment analyses, multiple testing corrections were applied using Bonferroni or Benjamini–Hochberg (BH) methods; only statistically significant or borderline significant associations were reported. For LDAK gene-based heritability estimation, the *P* value is calibrated using 10 permutations (default setting in LDAK5.2). In the GIANT analysis, enriched biological processes and pathways were considered significant at *P* < 0.05. For the functional module analysis, Q-values of each term were calculated using a one-sided Fisher’s exact test and BH corrections for multiple testing, as implemented in humanbase. The Enrichr Q-value represents an adjusted *P* value calculated using the BH method.

## RESULTS

### GWAS of elite Jam, A-A and Jpn athletes

In the discovery phase, following initial QC checks on DNA quantity and quality, GWAS genotype data were available for 95 Jam athletes and 102 Jam controls, 108 A-A athletes and 397 A-A controls, and 54 Jpn athletes and 118 Jpn controls. All athletes were specialist sprint or power-oriented athletes with ancestry-matched controls. Following standard GWAS QC procedures, a total of 609,801, 637,991 and 541,179 markers were retained in the Jam (88 athletes and 87 controls), A-A (79 athletes and 391 controls) and Jpn (54 athletes and 116 controls) cohorts. Seventeen, seven and thirty-six markers met a suggestive threshold of *P* = 5E-05 in these cohorts, respectively (Supplementary Table S2–S4). No variant exceeded the genome-wide significance threshold of *P* = 5E-08.

### Genotype imputation and meta-analyses

The IMPUTE2 pipeline identified 9,726,027 (Jam), 9,640,471 (A-A), and 6,444,519 (Jpn) markers, and the Sanger Imputation Server revealed 7,700,522 (Jam), 7,592,681 (A-A), and 4,975,382 (Jpn) markers (see Supplementary Fig. S3 and S4 for the GWAS and imputation Q-Q and Manhattan plots).

**FIG. 1:**
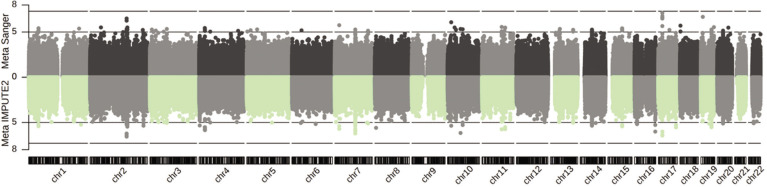
Genetic associations following imputation (IMPUTE2 and Sanger Imputation Server) and meta-analysis of Jam, A-A and Jpn cohorts with athletic prowess (sprinting) across 22 autosomes. Dotted line: suggestive significance cut-off of *P* = 1E-05; dashed line: genome-wide significance cut-off of *P* = 5E-08. X-axis: chromosome number; Y-axis: -log10(*P*).

The meta-analysis results comprised 11,038,143 (IMPUTE2) and 8,787,208 (Sanger) genetic variants, among which 107 (IMPUTE2) and 78 (Sanger) attained the suggestive significance cut-off of GC-corrected *P* = 1E-05 across two or three cohorts (Figure 1, and Supplementary Data S1 and S2). The genomic inflation factor was close to 1 (0.9995, IMPUTE2; and 1.004, Sanger; Supplementary Fig. S5). Notably, the most significant combined effect across all three cohorts was observed for the imputed variant rs113303758 located in the intron of *GALNT13* on chromosome 2. This variant exhibited a MAF ranging from 0.06–0.35 and a combined odds ratio (OR) of 2.52 for the C-allele (*P*_IMPUTE2_ = 2.75E-07 GC-corrected, *I*^2^ = 0%, *P*_het_ = 0.63; and *P*_Sanger_ = 3.16E-07 GC-corrected, *I*^2^ = 0%, *P*_het_ = 0.64). It is also tightly linked (in LD) with the GWAS array variant rs10196189 (*r*^2^ = 0.97, *D*’ = 1; OR_IMPUTE2_ = 2.46, *P*_IMPUTE2_ = 6.00E-07 GC-corrected, *I*^2^ = 0%, *P*_het_ = 0.70, and OR_Sanger_ = 2.48, *P*_Sanger_ = 5.45E-07 GC-corrected, *I*^2^ = 0%, *P*_het_ = 0.67, for the G-allele). The association results of rs113303758 and rs10196189 in individual cohorts following Sanger imputation are summarized in Table 1. At the cohort-specific level, the T-allele of rs117143557 (nearest gene: *MPRIP*, classified as a distal enhancer-like signature according to ENCODE [45]; MAF: 0.06) on chromosome 17 in Jpn had an OR of 17.05 (*P*_Sanger_ = 7.63E-08 GC-corrected), approaching the GWAS significance level of *P* = 5E-08 (Figure 1). In addition, the Sanger imputation results, which out-performed IMPUTE2, were used in the subsequent analyses detailed below.

**TABLE 1:** Association results of *GALNT13* rs10196189 and rs113303758 in individual GWAS cohorts following Sanger imputation.

CHR	SNP	BP	GWAS Cohort	Effect Allele	EAF	OR	95% CI	ln(OR)	SE ln(OR)	*P* *
2	rs10196189	154826491	Jam	G	0.36	2.83	1.77–4.53	1.04	0.24	1.69E-05
			A-A	G	0.32	1.99	1.13–3.52	0.69	0.29	0.02
			Jpn	G	0.06	2.66	1.04–6.83	0.98	0.48	0.04

2	rs113303758	154837893	Jam	C	0.35	2.92	1.82–4.67	1.07	0.24	9.91E-06
			A-A	C	0.32	2.01	1.14–3.56	0.70	0.29	0.01
			Jpn	C	0.06	2.64	1.03–6.76	0.97	0.48	0.04

CHR: chromosome; SNP: single nucleotide polymorphism; BP: base pair position, matching GRCh37; EAF: effect allele frequency; OR: odds ratio; 95% CI: 95% confidence interval; SE: standard error. Jam: Jamaicans, A-A: African-Americans and Jpn: Japanese.

* : Uncorrected *P* values.

### Replication of the GWAS array hit rs10196189 in athletes of European descent

In the replication phase, we investigated the top hit, rs10196189 (in almost perfect LD with rs113303758), and independently replicated its association with elite sprint and power performance in two elite European athlete-control cohorts. The first cohort comprised 234 power-oriented athletes, 451 endurance-oriented athletes and 1,525 controls from Belarus, Lithuania and Russia (OR_additive_ = 1.53, 95% CI = 1.17–2.00, *P* = 2.00E-03, for the G-allele of rs10196189 in the power-oriented athletes vs controls; OR_G-dominant_ = 1.61, 95% CI = 1.19–2.18, *P* = 1.80E-03; Table 2). The second cohort consisted of 171 sprint athletes, 252 endurance-oriented athletes and 595 controls from Australia, Belgium, Greece and Poland (OR_additive_ = 1.45, 95% CI = 1.05–2.00, *P* = 2.40E-02, for the G-allele of rs10196189 in the sprint athletes vs controls; OR_A-dominant_ = 0.28, 95% CI = 0.10–0.75, *P* = 7.00E-03; Table 2). Details on the replication cohorts for their geographical regions and genotype distribution within each cohort are provided in Supplementary Table S5 and S6. Moreover, meta-analysis combining the Sanger imputation and replication results for rs10196189 across the three GWAS cohorts and two replication cohorts revealed a significant association crossing the GWAS threshold of *P* = 5E-08 (OR_additive_ = 1.71, *P* = 2.28E-09; *I*^2^ = 41.7%, *P*_het_ = 0.16. When a sample-size weighted meta-analysis was performed, the significance remained robust (*P* = 2.13E-09; *I*^2^ = 47.8%, *P*_het_ = 0.11; Table 3).

**TABLE 2:** Replication results of *GALNT13* rs10196189 in two European cohorts

Replication Cohort	Sub Group	Effect Allele*; EAF	Additive Model		Dominant Model		

			OR^$^ (95% CI)	P^&^	Dominant Allele	OR^¶^ (95% CI)	P^&^
Cohort 1: Belarus, Lithuania, and Russia	Sprinters and Jumpers	(G); 0.14	1.23 (0.85–1.78)	0.27	G	1.32 (0.88–1.97)	0.17

	Other sprint/power oriented	G; 0.17	1.53 (1.17–2.00)	2.00E-03	G	1.61 (1.19–2.18)	1.80E-03

	Endurance oriented	(G); 0.13	1.10 (0.88–1.38)	0.42	A	0.74 (0.32–1.68)	0.47

Cohort 2: Australia, Belgium, Greece, and Poland	Sprinters and Jumpers	G; 0.19	1.45 (1.05–2.00)	2.40E-02	A	0.28 (0.10–0.75)	7.00E-03

	Other sprint/power oriented	(G); 0.14	1.02 (0.71–1.46)	0.92	A	1.13 (0.24–5.38)	0.88

	Endurance oriented	(A); 0.88	1.23 (0.89–1.70)	0.20	G	0.70 (0.49–1.01)	5.50E-02

EAF: effect allele frequency; OR: odds ratio; 95% CI: 95% confidence interval.

* : The allele whose frequency is higher in the athletes relative to the controls. It bears no meaning where the test results were non-significant (in brackets);

$ : Odds ratios are with respect to the effect allele;

¶ : Odd ratios are with respect to the dominant allele;

& : Uncorrected *P* values.

**TABLE 3:** Meta-analysis of association results for *GALNT13* rs10196189 across the GWAS imputation cohorts and the two European replication cohorts exceeding the genome-wide significance of *P* = 5E-08.

SNP	Effect Allele	OR	95% CI	P_combined_	Direction	I^2^	P_het_
rs10196189	G	1.71	1.43–2.04	2.28E-09	+++++	41.7%	0.14

When sample size weighted meta-analysis performed, *P*_combined_ = 2.13E-09, *I*^2^ = 47.8%, *P*_het_ = 0.11.							

SNP: single nucleotide polymorphism; OR: odds ratio; 95% CI: 95% confidence interval; *P*_combined_: combined fixed-effects meta-analysis *P* value based on the raw additive association *P* values from each cohort; *I*^2^: heterogeneity index (0–100%); *P*_het_: *P*value for heterogeneity.

### In silico search for rs10196189 and GALNT13 gene expression

Further *in silico* search for rs10196189 showed that the sprinting ability-increasing G-allele of rs10196189 was associated with higher *GALNT13* expression in the brain cortex (t-statistic = 2.2, *P* = 0.027; using the eQTL Calculator on https://gtexportal.org). In addition, the muscle biopsy analysis showed that *GALNT13* expression was positively associated with the relative area occupied by fast-twitch muscle fibers in the vastus lateralis muscle of 23 physically active men (t-statistic = -2.6, *P* = 0.018, adjusted for age, BMI and athletic levels).

### Gene- and gene-set enrichment analyses

In the gene-based analysis, 18,416, 18,414, and 18,317 genes containing at least one SNP were identified in the Jam, A-A and Jpn GWAS imputation cohorts, respectively. Following multiple testing correction, significant associations were observed for *BOP1* (*P*_multi_ = 6.52E-07, and *P*_snpwise_mean_ = 8.35E-07) and *HSF1* (*P*_multi_ = 1.71E-06, and *P*_snpwise_mean_ = 1.67E-06) in Jam, *GRM7* (*P*_multi_ = 1.28E-06) in A-A, and *MPRIP* (*P*_snpwise_top1_ = 1.63E-06) in Jpn. Furthermore, gene-level meta-analysis across the three cohorts revealed a significant association for *ZFYVE28* (*P*_multi_ = 2.01E-06) and a borderline significant finding for *ADAMTS18* (*P*_multi_ = 3.13E-06). Competitive gene-set enrichment analysis of these meta-analysis results identified 199 genes significantly enriched in interferon-gamma response, one of the 50 gene sets in the MSigDB Hallmark Collection [33] (*P* = 7.60E-04 after multiple testing correction; Supplementary Data S3). No significant enrichment was observed in any of the 2,922 Canonical pathways from the MSigDB C2 Collection.

### LDAK gene-based heritability estimation

LDAK gene-based heritability estimation for *MPRIP* in the Jpn cohort yielded an estimated heritability of 0.20 (SD: 0.09) on the observed scale, and 0.13 (SD: 0.06) on the liability scale, assuming a prevalence of 1% (test statistic = 20.87, *P*_permutation_ = 2.05E-06 following multiple testing correction). This significant finding aligns with that of the gene analysis results for Jpn mentioned earlier. Furthermore, the top gene unveiled in the Jam cohort is *STXBP2*, with an estimated heritability of 0.13 (SD:0.07) on the observed scale and 0.07 (SD: 0.04) on the liability scale (test statistic = 17.83, *P*_permutation_ = 6.91E-06). For the next two top genes, *BOP1* and *HSF1*, the estimated heritability on the observed scale is 0.10 (SD: 0.07) and 0.09 (SD: 0.09), respectively, while on the liability scale, the values are 0.06 (SD: 0.04) and 0.05 (0.04) (test statistic = 17.71, *P*_permutation_ = 7.38E-06 for *BOP1*, and test statistic = 16.83, *P*_permutation_ = 1.08E-05 for *HSF1*). These results are consistent with the gene analysis findings in Jam. *GALNT13*, harbouring the replicated rs10196189, showed a heritability estimate of 0.19 (SD: 0.08) on the observed scale and 0.11 (SD: 0.04) on the liability scale in Jam (test statistic = 9.14, *P*_permutation_ = 7.81E-04). In contrast, negligible heritability estimates were observed in A-A (test statistic = 0.02, *P*_permutation_ = 0.32) and Jpn (test statistic = 0, *P*_permutation_ = 0.68). It should be noted that these findings in Jam were not statistically significant after accounting for the number of genes analyzed.

### Tissue-specific functional network analysis (GIANT)

**FIG. 2:**
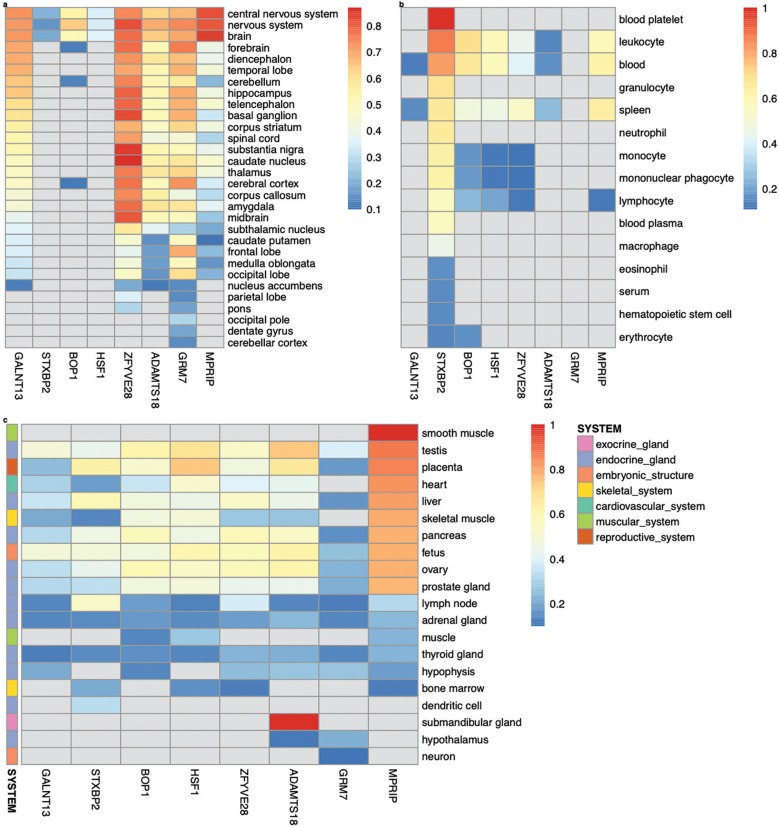
Tissue-/cell-type specific expression of *GALNT13, STXBP2, BOP1, HSF1, ZFYVE28, ADAMTS18, GRM7* and *MPRIP* in the nervous system (a), hematopoietic system (b), as well as other multiple systems (c), following the functional networks analysis in GIANT [37] (the data available under a CC-BY 4.0 license, see: https://humanbase.readthedocs.io/en/latest/).

Among the genes analyzed in GIANT *– GALNT13, STXBP2, BOP1, HSF1, GRM7, MPRIP, ZFYVE28* and *ADAMTS18 –* tissue-/cell-type specific expression networks revealed distinct patterns of expression. *GALNT13, GRM7, MPRIP, ZFYVE28,* and *ADAMTS18* are expressed almost universally across the nervous system (Figure 2a). Notably, *ZFYVE28* demonstrates the highest predicted confidence score for its expression in caudate nucleus (score: 0.87, Figure 2a). In contrast, *STXBP2* shows broad expression across the hematopoietic system (confidence scores ranging from 0.15 to 0.87), and is experimentally validated in blood platelet achieving the maximum confidence score of 1 (Figure 2b). Intriguingly, in addition to the nervous system, *MPRIP* is expressed across diverse tissue systems, including the muscular, endocrine, cardiovascular, skeletal, and embryonic systems. Its expression is experimentally validated specifically in smooth muscle (Figure 2c).

### Polypeptide N-acetylgalactosaminyltransferase 13 (GALNT13)

Tissue-specific gene expression of *GALNT13* was observed in the central nervous system, with its highest predicted confidence score of 0.73 relative to other nervous tissue types (Figure 2a). Within this network, 14 genes were identified as functionally similar to *GALNT13* based on interaction scores exceeding 0.15, including *GNAO1, ASIC1, GRIK2, MED12L, SORCS1, GRIK3, FZD4, SEZ6L, MYO7A, SHANK2, KMT2A, KANSL1, DSCAM*, and *PIEZO2* (Supplementary Fig. S6). Of these, seven genes were associated with key biological processes represented in the network: the nervous system process (*ASIC1, GRIK2, MYO7A* and *SHANK2*), sodium ion transmembrane transport (*ASIC1, GRIK2*, and *MYO7A*), histone H4–K16 acetylation (*KMT2A* and *KANSL1*), ionotropic glutamate receptor signalling pathway (*GRIK2* and *GRIK3*), and monovalent inorganic cation transport (*ASIC1, GRIK2* and *GRIK3*) (*P* < 3.70E-02). Most gene pairs associated with *GALNT13* in this network also show evidence supporting their role in the locus ceruleus (a nucleus in the pons of the brain stem, which activates the sympathetic nervous system). Interaction scores for these gene pairs ranging from 0.36 to 0.91(Supplementary Data S4).

For biological process-specific gene interactions, the *GALNT13*– *TF* (transferrin) gene pair demonstrated the strongest association with forebrain development (highest interaction score: 0.89). This was followed by other notable gene pairs, including *GALNT13*–*FUT6, GALNT13*–*SIX3, GALNT13*–*GCNT3, GALNT13*–*BTG4*, and *GALNT13*–*RTL3* with interaction scores ranging from 0.51 to 0.64 (Supplementary Fig. S6). In addition, key developmental process such as dorsal/ventral pattern formation, regionalization and pattern specification process are enriched in forebrain development (*P* < 3.80E-02), driven by network genes *SIX3* and *SMAD6*.

### Syntaxin binding protein 2 (STXBP2)

The highest predicted score for *STXBP2* was observed in leukocyte (score: 0.87, Figure 2b). Functionally similar genes to *STXBP2* include *CAPG, PTPN6, S100A11, EHBP1L1, IFI30, ARPC4, PTPN18, MVP, ACTN4, CYBA, TYMP, SBNO2, PFN1*, and *IKBKG*, all of which have interaction scores exceeding 0.46 (Supplementary Fig. S6). Gene pairs involving *STXBP2* in the network show evidence supporting their functions in osteoblasts, granulocytes (basophils), monocytes, dendritic cells, natural killer cells, locus ceruleus and dentate gyrus (interaction scores ranging from 0.51 to 0.93; Supplementary Data S4). Gene interactions specific to leukocyte migration are characterized by gene pairs *STXBP2*–*ARHGEF1, STXBP2*–*CNOT3, STXBP2*– *CAPG, STXBP2*–*SBNO2, STXBP2*–*GRN, STXBP2*–*CLDN4, STXBP2*– *MVP, STXBP2*–*STX4* and *STXBP2*–*POLD4* (interaction score ≥ 0.95; Supplementary Fig. S6). Notably, *STXBP2, SBNO2* and *STX4* are associated with several biological processes enriched in leukocyte migration, such as myeloid cell and leukocyte activation involved in immune response (*P* = 1.00E-03 and *P* = 4.00E-03, respectively), as well as granulocyte activation (*P* = 1.40E-02).

### BOP1 ribosomal biogenesis factor (BOP1) and heat shock transcription factor 1 (HSF1)

*BOP1* shows its highest expression in leukocyte (predicted score: 0.70), followed by blood (0.67) and the nervous system (0.64). Its expression is also observed in endocrine gland, such as testis (0.61), ovary (0.58), and pancreas (0.56) (Figure 2). *HSF1* is most expressed in the reproductive system (placenta, 0.74), followed by the endocrine gland (testis, 0.68; ovary, 0.54; prostate gland, 0.48; pancreas, 0.46; and liver, 0.45), embryonic structure (fetus, 0.60), leukocyte (0.58), blood (0.57), heart (0.52), skeletal muscle (0.49), and brain (0.36) (Figure 2). *BOP1* and *HSF1* are physically close and located on chromosome 8, with the strongest interaction confidence observed in the peripheral nervous system (0.97). This prediction is supported by evidence from gene co-expression (62%), transcription factor (TF) binding prediction (26%) and GSEA perturbations datasets (12%). Functionally similar genes in the network include *FGFR3, TRIP6, PTBP1, FGFR1, CCNF, UPF1, SLC52A2, SCRIB, GAL, C20orf27, NUP188, EIF3B* and *SMARCA4*, with interaction scores ranging from 0.88 to 0.96 (Supplementary Fig. S6). Among these genes, five are associated with viral gene expression (*PTBP1, EIF3B* and *SMARCA4*), IRES-dependent viral translation initiation (*PTBP1* and *EIF3B*), synostosis (*FGFR3* and *FGFR1*), and viral translation (*PTBP1* and *EIF3B*) enriched in the peripheral nervous system (*P* ≤ 2.80E-02). Gene pairs involving *BOP1* or *HSF1* in the network are also predicted to function in diverse tissues, including hepatocytes, corpus luteum, trophoblast, cardiac muscle, adrenal cortex, and the uterine cervix (interaction scores: 0.56 to 1.00; Supplementary Data S4).

In addition, *BOP1* and *HSF1* are specifically implicated in the ubiquitin-dependent protein catabolic process (Supplementary Fig. S6). The top three genes connected to *BOP1* and *HSF1*, i.e., *ARHGDIA, EIF4G1* and *ATP5F1D* (interaction score > 0.90), show experimentally validated associations in glutamate receptor signalling, ribonucleoprotein complex assembly, translation initiation, cellular respiration and rRNA processing. *BOP1* and *DHX30* are also associated with ribosomal large subunit assembly (*P* = 1.50E-02) and ribosomal biogenesis (*P* = 2.40E-02), processes that are enriched within the ubiquitin-dependent protein catabolic process.

### Glutamate metabotropic receptor 7 (GRM7)

*GRM7* is universally expressed across the nervous tissue system, with expression levels ranging from the lowest in parietal lobe, nucleus accumbens and cerebellar cortex (predicted score: 0.14) to the highest in the forebrain (0.76) (Figure 2a). In the forebrain, functionally similar genes to *GRM7* include *ATP2B2, MYT1L, GRIK3, LRP6, EDA, PKNOX2, CACNA1I, CATSPER2, NELL1, AFF2, GABRA4, DOCK1, PAPPA2*, and *RALYL* (interaction scores ranging from 0.28 to 0.41) (Supplementary Fig. S6). Among these genes, *GRM7* and *GRIK3* are present in several biological processes enriched in the forebrain network, particularly in neurotransmission pathways, such as the adenylate cyclase-inhibiting G-protein coupled glutamate receptor signalling pathway (*P* = 7.50E-04) and the G-protein coupled receptor signalling pathway, coupled to cyclic nucleotide second messenger (*P* = 4.40E-02). In addition, *GRM7* and *ATP2B2* are associated with sensory perception of sound and mechanical stimulus (*P* = 2.20E-02). The majority of gene pairs involving *GRM7* in the network are also supported by evidence for their role in locus ceruleus (interaction scores ranging from 0.68 to1; Supplementary Data S4).

For biological process-specific gene interactions in the glutamate receptor signalling pathway, the genes *GRM7, GRM1, GRIK3, GRIK1, GRIA1, GRIA2, GRIN1*, and *GRMB* are over-represented in this network (*P* = 1.00E-12). Furthermore, *GRIK3, GRIK1, GRIA1, GRIA2*, and *GRIN*1 associate with ionotropic glutamate receptor signalling pathway (*P* = 5.20E-09), *GRM1, GRM7, GRM8*, and *GRIK3* with G-protein coupled glutamate receptor signalling pathway (*P* = 6.30E-09), and *GRM7, GRM8* and *GRIK3* with adenylate cyclase-inhibiting G-protein coupled glutamate receptor signalling pathway (*P* = 2.60E-07), highlighting the integral role of these genes in glutamate receptor signalling (Supplementary Fig. S6).

### Myosin phosphatase Rho interacting protein (MPRIP)

*MPRIP* is expressed across multiple tissue systems, particularly in smooth muscle, endocrine gland (e.g., testis, liver, pancreas, ovary, and prostate gland), placenta, skeletal muscle and the nervous system (Figure 2). In these tissue networks, *MPRIP* and *YWHAB* strongly interact in smooth muscle (interaction score: 0.71), testis and placenta (0.82), skeletal muscle (0.73), and brain (0.61). This genepair is also predicted to function in spermatogonium (0.96), parietal lobe (0.95), pons (0.93), eosinophil (0.92), spermatid (0.92) and T-lymphocyte (0.89). In addition, *MPRIP* and *YWHAB* participate in a variety of specific biological processes and pathways, with interaction scores ≥ 0.9. These processes include, in descending order of interaction score, translational initiation, gene silencing by RNA, RNA splicing, cell adhesion mediated by integrin, cellular senescence, actin cytoskeleton reorganization, acute inflammatory response, T cell proliferation, cell morphogenesis, erythrocyte differentiation, cardiac muscle contraction, extracellular matrix organization, telomere maintenance, Ras protein signal transduction, neuron projection morphogenesis, response to hypoxia, chemical synaptic transmission, necrotic cell death, angiogenesis, actin filament bundle assembly, T-cell differentiation, T-cell mediated immunity, and response to virus.

Other genes functionally similar to *MPRIP* show enrichment in the following biological processes. *PPM1F* and *ITGB1BP1* are associated with cell-substrate adherens junction assembly in the brain (*P* = 2.90E-02), *ITGB1BP1* and *TAX1BP3* with negative regulation of cellular protein localization in the brain (*P* = 3.90E-02), *LAMC1, RHOA* and *MACF1* in cell junction assembly and cell junction organization in skeletal muscle (*P* = 3.40E-02), and *RHOA, MACF1* and *YWHAG* in regulation of neuron differentiation, regulation of neurogenesis and regulation of nervous system development in skeletal muscle (*P* ≤ 4.60E-02).

### GALNT13, GRM7 and MPRIP

These three genes originate from the Jam (*GALNT13*), A-A (*GRM7*) and Jpn (*MPRIP*) GWAS cohorts, crossing the suggestive threshold of *P* = 5E-05. Notably, they are almost universally expressed across the entire nervous tissue system (Figure 2a). Given their widespread expression, we focused our analysis solely on their interactions within the nervous system. They are most strongly characterized in the glutamate receptor signalling pathway. To refine the gene network, we applied a minimum interaction score of 0.20, limiting analysis to the top 10 genes unique to the query genes. These include *PCD-HGC5, OR4D2, TAS2R50, KNG1, ANKRD31, KRT82, TEX26, FOXR2, MGAT4C* and *EXOC3L4* (Supplementary Fig. S6).

*GALNT13* and *GRM7* have an interaction score of 0.33 within the glutamate receptor signalling pathway (interaction evidence: 79% co-expression, 20% GSEA perturbations, and 1% TF binding). However, their strongest interaction is observed in chemical synaptic transmission (interaction score: 0.80). Most gene pairs associated with *GALNT13* or *GRM7* show co-expression in the glutamate receptor signalling pathway. In addition, there is functional evidence supporting their roles in chemical synaptic transmission, detection of chemical stimulus, sodium ion transport, potassium ion transport, hormone secretion, cardiac ventricle development, cardiac muscle contraction, and the cAMP biosynthetic process (interaction scores ranging from 0.29 to 1.00; Supplementary Data S4). For *MPRIP,* direct interactions were identified with *ANKRD31, EXOC3L4* and *PCD-HGC5* in chemical synaptic transmission (0.26), cartilage development (0.47) and cardiac ventricle development (0.58), respectively; in addition to their functional relationship to glutamate receptor signalling pathway, with interaction scores of 0.27, 0.50 and 0.69, respectively (Supplementary Data S4).

### Zinc finger FYVE-type containing 28 (ZFYVE28)

Tissue-specific expression of *ZFYVE28* is prominent across the nervous system, with the highest predicted expression in the caudate nucleus (predicted score: 0.87), followed by the substantia nigra (0.85) and basal ganglion (0.83) (Figure 2a). In the caudate nucleus, *CHD9, PRNP, PSMB7, SUPT7L, USP46, ACOT11* and *TTC17* interact with *ZFYVE28*, with interaction scores ranging from 0.16 to 0.20 (Supplementary Fig. S6). These interactions are similarly observed in the amygdala. Other specific interactions include *PSMB7, USP46, ACOT11* with *ZFYVE28* in the substantia nigra (0.18–0.19), and *CHD9, PRNP, TTC17* with *ZFYVE28* in the basal ganglion (0.18–0.21). Most of these gene pairs show stronger interactions in the locus ceruleus, with scores ranging from 0.29 to 0.72 (Supplementary Data S4).

### ADAM metallopeptidase with thrombospondin type 1 motif 18 (ADAMTS18)

*ADAMTS18* is expressed in the submandibular gland (Figure 2c) and ubiquitously across the nervous tissue system, with its highest expression observed in the nervous system (predicted score: 0.69, Figure 2a). Top genes functionally similar to *ADAMTS18* in the nervous system include *INHBB, SLC9A2, ITGA11, BCL2, SMOC2, CSMD1, BAIAP2, PDGFRA, JPH3, ACTL6B, WNT1, TBC1D26, CHRD* and *GDF6* (interaction scores ranging from 0.16 to 0.19, Supplementary Fig. S6). Key biological processes associated with *ADAMTS18* and its network genes include: *INHBB, PDGFRA, WNT1* and *AD-AMTS18* in response to wounding (*P* = 2.10E-03), *INHBB, WNT1, CHRD* and *GDF6* in transmembrane receptor protein serine/threonine kinase signalling (*P* = 2.10E-03), *WNT1, CHRD* and *GDF6* in BMP signalling pathway and cellular response to BMP stimulus (*P* = 2.10E-03), *ADAMTS18* and *PDGFRA* in negative regulation of platelet activation (*P* = 2.10E-03) and aggregation (*P* = 6.10E-03), *PDGFRA, WNT1, CHRD* and *GDF6* in cellular response to growth factor stimulus (*P* = 6.10E-03), *PDGFRA, WNT1, ADAMTS18*, and *BAIAP2* in cell-cell adhesion (*P* = 6.10E-03), *PDGFRA* and *WNT1* in positive regulation of fibroblast proliferation (*P* = 8.60E-03), *PDGFRA, BCL2*, and *ADAMTS18* in regulation of cell activation (*P* = 1.30E-02) and *WNT1* and *GDF6* in skeletal system development (*P* = 4.70E-02). Four gene pairs, *ACTL6B*–*ADAMTS18, JPH3*–*ADAMTS18, TBC1D26*– *ADAMTS18* and *CSMD1*–*ADAMTS18,* also closely interact in the locus ceruleus with interaction scores of 0.24, 0.51, 0.63 and 0.88, respectively (Supplementary Data S4).

### Blood-specific functional modules

**FIG. 3:**
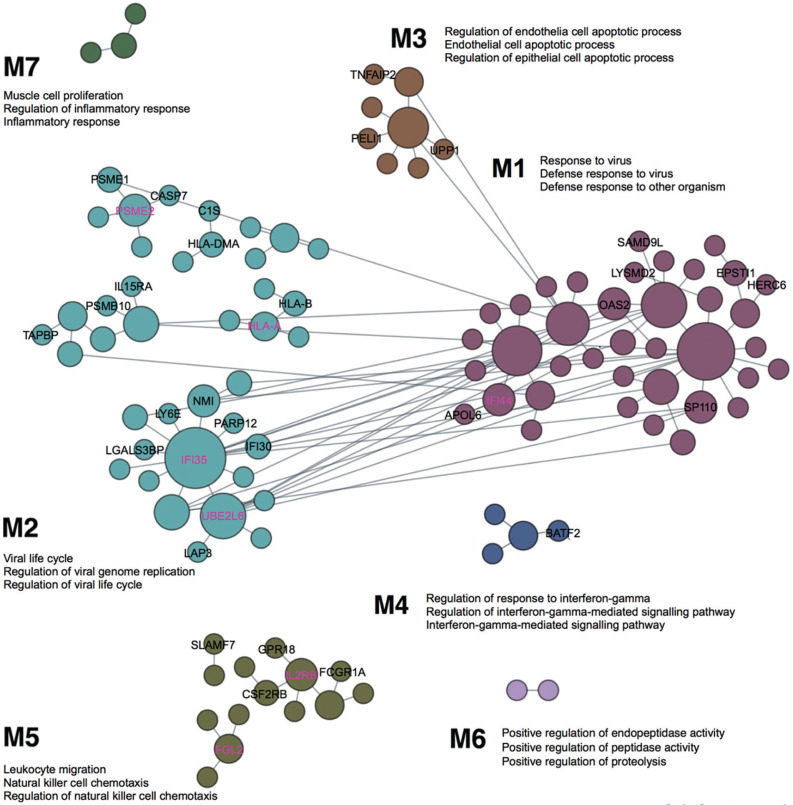
Cellular responses to interferon-gamma unveiled in blood-specific functional modules, linking individual genes to specific biological processes. Top three enriched GO terms are annotated for each module, and novel, previously uncharacterized genes are labelled in M1-M7 (among which, the highest ranked genes are marked in magenta based on the number of edges they associate with). The figure is adapted from the output of the modules analysis implemented in humanbase [40] (the data available under a CC-BY 4.0 license, see: https://humanbase.readthedocs.io/en/latest/)

The previous competitive gene-set enrichment analysis revealed 199 genes enriched in the interferon-gamma response gene-set. We now focus on uncovering blood-specific functional modules within this gene set. Of the 197 genes (excluding *WARS1* and *MARCHF1* not matched in the humanbase data collections), 109 genes were categorized into specific modules (M1–M7, Figure 3). The top three representative biological processes, annotated to Gene Ontology terms, in each module, consist of response to virus, defense response to virus, and defense response to other organism (M1, 38 genes; Q < 1.00E-4); viral life cycle, regulation of viral genome replication, and regulation of viral life cycle (M2, 40 genes; Q < 1.00E-4); regulation of endothelial cell apoptotic process, endothelial cell apoptotic process and regulation of epithelial cell apoptotic process (M3, 8 genes; Q < 1.00E-4); regulation of response to interferon-gamma, regulation of interferon-gamma-mediated signalling pathway, and interferon-gamma-mediated signalling pathway (M4, 4 genes; Q < 1.00E-4); leukocyte migration, natural killer cell chemotaxis, and regulation of natural killer cell chemotaxis (M5, 15 genes; Q = 1.00E-4); positive regulation of endopeptidase activity, positive regulation of peptidase activity, and positive regulation of proteolysis (M6, 2 genes; Q ≤ 8.00E-4); and muscle cell proliferation, regulation of inflammatory response and inflammatory response (M7, 3 genes; Q ≤ 8.00E-4). Among the 109 genes, 8, 18, 3, 1, and 6 genes are uncharacterized in M1–M5, respectively (36 genes in total); in other words, approximately 21% to 43% of genes in M1–M5 lack annotation to specific Gene Ontology terms, suggesting their potentially novel roles within these specific functional modules (Figure 3). Notably, *IFI44* is the top-ranked uncharacterized gene in M1, while *IFI35, PSME2, HLA-A* and *UBE2L6* are top-ranked in M2, and *IL2RB* and *FGL2* in M5 (Figure 3). The uncharacterized pairs *PSME2*–*PSME1* and *HLA-A*–*HLA-B* in M2 display strong edge weights of 0.997 and 0.987, respectively. In addition, *IFI35* in M2 is associated with 11 other genes in the local network, including 6 uncharacterized genes (*IFI30, LGALS3BP, LY6E, NMI, PARP12* and *UBE2L6*; Figure 3). The edge weights for these interactions range from 0.981 (*IFI35*–*ISG15*) to 0.816 (*IFI35*–*MVP*) (Supplementary Data S5).

### Genomic variant effect analysis (Sei analysis)

The variant effects of six sentinel SNPs, representing independent regional signals near or within *GLANT13, STXBP2, BOP1, GRM7, MPRIP, CERS4*, on tissue- and cell-type-specific regulatory activities were quantified in Sei (Table 4). These six SNPs were identified from the discovery GWAS, crossing the suggestive cut-off of *P* = 5E-05 (rs2303115, *STXBP*, Jam) or were replaced by stronger Sanger imputation signals in almost perfect LD: rs4977199, (*BOP1, P* = 7.73E-07, Jam) and rs2875287 (*GRM7, P* = 3.79E-07, A-A). They also include top associations following genotype imputation and meta-analysis: rs117143557 (*MPRIP*, Jpn, *P*_GC-corrected_ = 7.63E-08), rs2927712 (*CERS4* across Jam and A-A, *P*_GC-corrected_ = 2.08E-07), and rs113303758 (*GALNT13* across Jam, A-A, and Jpn, *P*_GC-corrected_ = 3.16E-07).

**TABLE 4:** Tissue-/cell-type-specific regulatory activities associated with sentinel SNPs resided within or next to genes derived from the SNP- and gene-based analysis in Sei [39] (predictions made for 40 sequence classes and 21,907 regulatory features; the data available under a CC-BY 4.0 license, see: https://humanbase.readthedocs.io/en/latest/).

Gene	rs ID	CHR:BP	Ref Allele	Alt Allele	OR (95% CI); cohort(s)	Maximum sequence-class score
*GALNT13*	rs113303758	2:154837893	T	C	2.52 (1.77–3.58); across Jam, A-A and Jpn	-0.022
*GRM7*	rs2875287	3:7676406	G	A	4.99 (2.68–9.28); A-A	-0.028
*BOP1*	rs4977199	8:145508691	C	G	0.35 (0.23–0.53); Jam	-0.84
*MPRIP*	rs117143557	17:16933431	C	T	17.05 (6.19–46.98); Jpn	-0.085
*STXBP2*	rs2303115	19:7708214	G	A	2.80 (1.80–4.36); Jam	0.21
*CERS4*	rs2927712	19:8333905	A	C	0.20 (0.11–0.37); across Jam and A-A	1.05


**Gene**	**Corresponding sequence class**			**Representative regulatory features (Alt-Ref allele probability diff score)**		
*GALNT13*	HET2 Heterochromatin			Blast Cells Blood / H3K9me2 (0.027)		

*GRM7*	HET5 Centromere			Jurkat T Lymphocyte Blood / TET2 (-0.13)		

*BOP1*	E4 Multi-tissue			ECC-1 Epithelium Endometrium / NFIC (-0.92); K562 Erythroblast Bone Marrow / NFIA (-0.82); SK-N-SH RA Neuron Brain / DNase (-0.50); Neuroblastoma cell / H3K4me1 (-0.32)		

*MPRIP*	PC1 Polycomb / Heterochromatin			Kidney / RUNX1 (-0.061)		

*STXBP2*	TN1 Transcription			H1 Derived Mesenchymal Stem Cells / H3K36me3 (0.13); CD19 Primary Cells Peripheral / H3K36me3 (0.12)		

*CERS4*	TF3 FOXA1 / AR / ESR1			VCaP Epithelium Prostate / AR (0.21); Astrocyte Brain / H3K9ac (0.16); Jurkat T Lymphocyte Blood / ATAC-seq (0.16)		

rs ID: SNP reference id; CHR: BP: chromosome and base pair position, matching GRCh37. ORs are relative to the Ref allele. Jam: Jamaicans, A-A: African-Americans and Jpn: Japanese.

### Sequence class-level variant effects

The C-allele of rs2927712 (an intergenic variant, nearest *CERS4*) increases the activity of TF sequence classes, notably TF3 FOXA1/AR/ESR1 (maximum sequence class score: 1.05), TF5 AR (0.97), as well as the enhancer (E) class E4 Multi-tissue (0.72). In contrast, the C-allele decreases the activity of E5 B-cell-like (-0.50). The A-allele of rs2303115 (intron variant in *STXBP2*) increases the activity of transcription (TN) sequence classes TN1 (0.21), TN2 (0.18) and TN4 (0.17). Meanwhile, the G-allele of rs4977199 (intron variant in *BOP1*) mitigates the activity of several enhancer classes, including E4 Multi-tissue (-0.84), E3 Brain/Melanocyte (-0.64), and E1 Stem cell (-0.62), P promotor (-0.56), and TF3 FOXA1/AR/ESR1 (-0.50), while enhancing transcription in sequence classes TN1 (0.70), TN4 (0.69) and TN2 (0.45).

The C-allele of rs113303758 (intron variant in *GALNT13*) overall decreases the activity of heterochromatin (HET) sequence classes HET2 (-0.022) and HET1 (-0.020). The A-allele of rs2875287 (intron variant in *GRM7*) decreases the activity of HET5 Centromere (-0.028) and HET6 Centromere (-0.018), while increasing the activity of HET4 heterochromatin (0.019) and HET3 heterochromatin (0.015). The T-allele of rs117143557 (an intergenic variant, nearest *MPRIP*) results in a global reduction of activity in all sequence classes, particularly in the Polycomb (PC) sequence class PC1 Polycomb/Heterochromatin (-0.085).

### Regulatory feature scores for 21,907 chromatin profiles

The C-allele of rs2927712 shows a higher probability of increasing androgen receptor activity in VCaP Epithelium Prostate (maximum alt-ref allele diffs score: 0.21), H3K9ac-marked transcription activity in Astrocyte Brain (0.16) and chromatin accessibility in Jurkat T Lymphocyte Blood (0.16). The A-allele of rs2303115 exhibits a higher probability of enhancing H3K36me3-marked transcription activity in Fetal Stomach (0.18), Fetal Intestine Small (0.15), Fetal Muscle Leg (0.14), H1 Derived Mesenchymal Stem Cells (0.13), CD19 Primary Cells Peripheral (0.12), Monocytes-CD14+ RO01746 (0.11). The G-allele of rs4977199 decreases the activities of *NFIC* in ECC-1 Epithelium Endometrium (-0.92), *NFIA* in K562 Erythroblast Bone Marrow (-0.82), DNase in SK-N-SH RA Neuron Brain (-0.50) and heart (-0.35), and H3K4me1-marked enhancer activity in Neuroblastoma cell (-0.32) and Brain Cingulate Gyrus (-0.16). It increases the activities of *CNOT3* in HCT-116 Colorectal cancer cell line (0.20), *CBFB* in ME-1 Leukaemia cell (0.13), H3K36me3-marked transcription in Left Ventricle (0.12) and Adipose Nuclei (0.12), and H3K-27me3-marked repression in Astrocyte (0.12).

The C-allele of rs113303758 is linked to H3K9me2-marked epigenetic repression in Blast Cells Blood (0.027) and K562 Erythroblast Bone Marrow (0.019). The A-allele of rs2875287 is associated with the reduced activities of *TET2* in Jurkat T Lymphocyte Blood (-0.13) and H3K27me3-marked repression in PAR Astrocyte (-0.091), while increasing H3K27me3 activity in Jurkat T Lymphocyte Blood (0.060). The T-allele of rs117143557 has a variety of functional roles, such as associations with H3K-4me3-marked promoter activity in IMR90 Fibroblast Lung (0.060), H3K27ac-marked enhancer activity in Monocyte Blood (0.055), H3K27me3-marked repression in Proerythroblast Bone Marrow (0.041), and H3K18ac-marked DNA demethylation [46] in H1 Derived Neuronal Progenitor Cultured Cells (0.038). Conversely, it decreases the activities of the transcription factor RUNX1 in kidney (-0.061) and repressive H3K27me3 marks in PAR Astrocyte (-0.051).

### GWAS reprioritization (NetWAS) and enrichment analysis in Enrichr

Using the central nervous system network, the 14,788 genes reprioritized with NetWAS from the Jam cohort show significant enrichment in the HDSigDB Human 2021 gene sets. For example, these include top neuron-specific and neuron-enriched genes in humans and mice (OR = 1.96, Q = 7.64E-13, neuron-specific and OR = 1.82, Q = 1.55E-10, neuron-enriched), genes changed in striatum and cortex of HdhQ175 mice (OR = 1.45, Q = 4.46E-11 striatum and OR = 1.52, Q = 1.65E-08 cortex), H3K27me3-enriched genes in MSNs of adult mice (OR = 1.43, Q = 5.29E-08) and differentially expressed genes in hippocampus of Foxp1 knock-out versus wild-type mice (OR = 1.40, Q = 1.29E-07). A full list of enrichment terms, overlapping genes, raw P-value, Q-value, OR and Enrichr combined score for the HDSigDB Human 2021 is provided in Supplementary Data S6. Furthermore, the reprioritized genes are highly expressed across a range of brain regions and the spinal cord in the ARCHS4 Tissues gene sets (Supplementary Data S7). Significant enrichment is also observed in glutamatergic neurons from the CellMarker Augmented 2021 gene-set library (OR = 6.66, Q = 5.00E-05) and in the adult frontal cortex gene-set from ProteomicsDB (OR = 1.97, Q = 6.00E-05).

In the forebrain network, the 15,472 genes reprioritized from the A-A cohort are enriched for developmental transcription factor genes bound by Suz12 (OR = 1.89, Q = 4.10E-02), which regulate early developmental processes, such as neurogenesis, haematopoiesis and cell-fate specification, in HDSigDB Human 2021 (Supplementary Data S8). These genes also show enrichment for expression in macrophages (OR = 2.44, Q = 1.60E-03), dendritic cells (OR = 2.37, Q = 1.66E-03), hematopoietic stem cells (OR = 2.09, Q = 7.58E-03), microglia (the resident macrophage cells of the brain and spinal cord; OR = 2.15, Q = 9.53E-03) and GABAergic neurons (OR = 2.23, Q = 2.65E-02) from the single-cell RNA-seq database PanglaoDB (Supplementary Data S9).

Using the brain network, 1,846 genes reprioritized from the Jpn cohort are enriched for NP2 neural progenitor cell-enriched genes (OR = 1.64, Q = 4.10E-17) and genes down-regulated in Sdt/Npy-expressing interneurons of HD patients versus controls (OR = 1.62, Q = 2.90E-16) in the HDSigDB Human 2021 (Supplementary Data S10). These reprioritized genes also overlap significantly with genes expressed in prefrontal cortex (OR = 1.76, Q = 1.10E-03), CD8+ T cells (OR = 1.53, Q = 2.00E-02) and cerebellum peduncles (OR = 2.21, Q = 2.00E-02) in the Human Gene Atlas gene-sets (Supplementary Data S11). Further enrichment is observed in fibrous astrocytes (Human Astro L1-6 FGFR3 AQP1 down, OR = 2.08, Q = 1.99E-08) from the Allen Brain Atlas 10x scRNA 2021 library (Supplementary Data S12) and in neurons from the retrosplenial area, lateral agranular part, and layer 6b (OR = 2.03, Q = 3.40E-02) from Allen Brain Atlas (Supplementary Data S13).

Using the tissue network for the caudate nucleus, 13,301 genes reprioritized from the gene-level meta-analysis results are also enriched for top neuron-enriched and neuron-specific genes in humans and mice (OR = 1.52, Q = 8.75E-06, neuron-enriched and OR = 1.36, Q = 4.60E-03 neuron-specific), and for those altered in brain versus spinal cord derived oligodendrocyte progenitor cells (OPCs) (OR = 1.41, Q = 2.60E-03), up-regulated in hippocampus of Foxp1 knock-out versus wild-type mice (OR = 1.29, Q = 2.70E-04) and up-regulated in Pvalb/Th-expressing interneurons of zQ175DN versus WT (OR = 2.22, Q = 8.40E-03) in the HDSigDB Human 2021 gene sets (Supplementary Data S14). This gene list also includes genes highly expressed across the whole brain, superior frontal gyrus, prefrontal cortex, motor neuron, cerebral cortex and the spinal cord in the ARCHS4 Tissues gene sets (Supplementary Data S15). It also overlaps with genes expressed in layer 1 of the AOV cortex, layer 1 of the frontal cortex (FCx), mantle zone of AOV, superficial stratum of AOV, Forel’s field, and nucleus of the posterior commissure in the Allen Brain Atlas gene-sets (Supplementary Data S16) and in the human motor cortex in the HuBMAP ASCTplusB augmented 2022 gene-sets (Supplementary Data S17).

Additionally, focusing on the four genes with the highest positive NetWAS score – ubiquinol-cytochrome c reductase, Rieske iron-sulfur polypeptide 1 (*UQCRFS1*; Jam), protein tyrosine phosphatase non-receptor type 6 (*PTPN6*; A-A), RALY heterogeneous nuclear ribonucleoprotein (*RALY*; Jpn), and zinc finger MYM-type containing 4 (*ZMYM4*; meta-analysis) (Supplementary Data S18) – a knowledge search in Enrichr gene-set libraries highlights their potential functional relevance. Knockdown of *UQCRFS1* results in extended life span in female mice (MP:0001661) and in contrast, leads to premature death in male mice (MP:0002083) in MGI Mammalian Phenotype Level 4 2021. *UQCRFS1* is also down-regulated in the FCx during aging in humans (GSE53890), rats (GDS3939) and mice (GSE20411) in the Aging Perturbations from GEO libraries. Knockdown of *PTPN6* is linked to abnormal astrocyte morphology (MP:0002182), forebrain morphology (MP:0000783), and nervous system morphology (MP:0003632), as well as decreased brain size (MP:0000774), abnormal hippocampus development (MP:0000808), and reduced neuron number (MP:0008948). Other associated phenotypes include decreased apoptosis (MP:0006043), decreased bone mass (MP:0004016), and increased erythroid progenitor cell number (MP:0003135) in the MGI Mammalian Phenotype Level 4 2021. Knockdown of *RALY* results in abnormal lung morphology (MP:0001175) and shortened tibia length (MP:0002764) in the MGI Mammalian Phenotype Level 4 2021. Furthermore, *RALY* is up-regulated in Claustrum cells and in the primary visual area, layer 5 cells in Allen Brain Atlas gene-sets. Knockdown of *ZMYM4* results in decreased body length (MP:0001258), decreased lean body mass (MP:0003961), and reduced mean platelet volume (MP:0008935) in the MGI Mammalian Phenotype Level 4 2021. *ZMYM4* is also down-regulated in Alzheimer’s Disease (GSE1297), Parkinson’s Disease (GSE7621), chronic obstructive pulmonary disease (GSE3320) and large granular lymphocytic leukaemia (GSE10631) in the Disease Signatures from GEO 2014.

## DISCUSSION

Here, we conducted a cross-ancestry, multi-phase GWAS in world-class sprint and power athletes and their matched controls. We carried out both the SNP- and gene-based analyses, complemented by machine-learning based functional characterization and tissue-specific reprioritization of GWAS associations. Specifically, genotype imputation enhanced the strength of GWAS findings despite the limited sample size (Supplementary Fig. S4), uncovering stronger and novel associations for *GALNT13, BOP1, HSF1, GRM7, MPRIP* and *STXBP2* (Supplementary Figures S7–S11). Replication and meta-analysis further boosted the power for the detection of genetic associations for *GALNT13, ZFYVE28, ADAMTS18* and *CERS4*. Functional characterization provided key insights into the regulatory roles of specific variants: rs4977199 in *BOP1*, rs2303115 in *STXBP2* and rs2927712 in *CERS4*, which were found to modulate enhancer activity, transcription, and transcription factor activity, respectively (Table 4). Functional predictions also revealed 36 previously uncharacterized genes implicated in host defence, leukocyte migration, and cellular responses to interferon-gamma (Figure 3). Furthermore, four genes–*UQCRFS1, PTPN6, RALY* and *ZMYM4*–were identified for their associations with traits such as aging, forebrain and nervous system morphology, lung and bone morphology, neurodegenerative diseases and blood disorders.

Across ancestries, the most striking result is the overrepresentation of the G-allele of rs10196189 (*GALNT13*) in elite sprint and power athletes of West African, East Asian and European descent, crossing the GWAS significance threshold of *P* = 5E-08 (Table 3). The G-allele of rs10196189 is associated with higher *GALNT13* expression in the brain cortex (data from the GTEx portal). In addition, the *GALNT13* expression is positively associated with the relative area occupied by fast-twitch muscle fibers observed in the current muscle biopsy analysis. Consistent with these findings, previous studies report that *GALNT13* is predominantly expressed in the central nervous system and may promote neurogenesis [47]. Collectively, these results suggest a regulatory role of rs10196189 in muscle fiber innervation and specification [48]. Moreover, gene-based analysis unveiled statistically significant associations for *ZFYVE28* and borderline significance for *ADAMTS18*, across the West African and East Asian GWAS cohorts. All three genes, *GALNT13, ZFYVE28* and *ADAMTS18*, are universally expressed in the nervous tissue system in GIANT analysis (Figure 2 and Supplementary Fig. S6). Notably, *ZFYVE28* is found to mediate insulin resistance, with higher expression levels in obese, non-diabetic patients (insulin resistant individuals) [49]. Further, *Zfyve28* overexpression in mice impaired insulin sensitivity and increased lipid content in the serum and liver [49]. Consistent with the current findings on *ZFYVE28*, previous studies have reported that elite power athletes exhibit greater insulin resistance than elite endurance athletes [50], highlighting distinct metabolic characteristics among athletes and patient populations. The beneficial and detrimental effects of insulin resistance require further investigation to improve athletic performance while minimising risks for type 2 diabetes and heart disease in both athletes and general population. *ADAMTS18* is critical for the development and morphology of epithelial organs, the vascular and neuronal systems, adipose tissue and reproductive tracts [51]. Aberrations in this gene have been linked to a range of pathological conditions, including cancers and eye disorders [51]. Intriguingly, *ADAMTS18* has been associated with bone mineral density (BMD) across three ethnic groups, including white U.S. families, a Chinese BMD sample, and a cohort of African ancestry. Its expression is significantly higher in subjects with normal-healing skeletal fractures than those with delayed healing [52]. In addition, senior Olympic athletes in high-impact sports exhibit higher BMD levels compared to those in non-high-impact sports [53]. Again, in line with the current findings on *ADAMTS18,* these data together suggest a pivotal role for *ADAMTS18* in maintaining bone health in athletes and serving as a potential therapeutic target for osteoporosis.

For cohort-specific findings, multiple lines of evidence highlight the significance of the findings for: *MPRIP* (T-allele of rs117143557; Jpn), *GRM7* (A-allele of rs2875287; A-A), *BOP1* (G-allele of rs4977199; Jam), *CERS4* (C-allele of rs2927712; Jam and A-A), and *STXBP2* (A-allele of rs2303115; Jam) (Table 4). These associations are supported by strong SNP- and gene-based signals or their regulatory effect scores in Sei. Intriguingly, rs4977199, rs2927712 and rs2303115, as the lead SNPs in their respective gene regions (*BOP1, CERS4, and STXBP2*), also act as statistically significant *cis*-eQTLs for other genes in blood-derived expression data [54], such as rs4977199 for *DGAT1* (encoding a key metabolic enzyme), rs2927712 for *CD320* (involved in B-cell proliferation) and rs2303115 for *PCP2* (predicted to locate in neuronal cell body). These data confirm that the three SNPs identified impact elite sprint and power performance via their regulatory roles on the transcriptome. This warrants further investigation into the shared genetic mechanisms underlying elite athletic ability, metabolic homeostasis, and normal immune and neuronal functions. These findings also offer translational potential for identifying new therapeutic targets in the relevant disease contexts. The T-allele of rs117143557 nearest *MPRIP* with a MAF exceeding 5% shows a large effect of on elite sprint and power performance in Jpn athletes (Table 4). In contrast, the T-allele is present at much lower frequencies in European and African populations (MAF < 1%), indicating a potential population-specific function. Further study is required to validate its target gene and understand its functional role in elite athletic status as studied here and fitness- and health-related phenotypes for this novel intergenic variant. The lack of functional evidence in Sei for the intronic variant rs2875287 in *GRM7* underscores the novelty of this finding. *GRM7* is known to regulate neurotransmission and mutations in this gene that result in reduced expression have been linked to neurodevelopmental disorders [55, 56], suggesting *GRM7* is a disease-causing gene. The association with elite sprint and power performance in the A-A cohort indicates a potential role for rs2875287 in maintaining neurological and cognitive functions in athletes. Further investigation is necessary to understand the variant effect on the glutamatergic pathway. These cohort-specific genes are specifically expressed in the nervous tissues system, except for *MPRIP* (expressed across multiple tissue systems, including the nervous tissues) and *STXBP2* (primarily in the hematopoietic system) (Figure 2 and Supplementary Fig. S6).

In addition to the above, the tissue-specific network analysis has provided exploratory links to the most relevant interacting genes in specific tissues and biological processes, as detailed in the GIANT section above. These predicated and functionally similar genes can be integrated into the broader biological contexts to refine and test new hypotheses for their roles in the nervous system, forebrain development, glutamate signalling pathway, higher neurological functions in caudate nucleus, and leukocyte migration (Supplementary Fig. S6).

Other notable findings from this study include the identification of 36 novel genes in populations of West Africans (Jam and A-A) and East Asians (Jpn) associated with specific functional modules indicative of response to virus (M1), viral life cycle (M2), cell apoptosis (M3), response to interferon-gamma (M4) and leukocyte migration (M5) (Figure 3). These results coincide with recent discoveries that *IFI44, OAS2*, and *HERC6* (in M1) are present in IFN responsive T cells in SARS-CoV-2 infected macaques, suppressing viral transcription and aiding clearance of the virus [57]. In addition, *IFI44* has been reported to repress HIV-1 replication *in vitro* [58]. Evidence for *NMI* and *IFI35* (in M2) indicates that they function as damage-associated molecular patterns in the extracelluar space, mediating proinflammatory responses to cellular infection and damage through the activation of the nuclear factor-κB in the Toll-like receptor 4 signalling pathway [59]. More recently, *IFI35* has been proposed as a biomarker for neuroinflammation and for predicting long-term treatment response in multiple sclerosis patients [60]. Autosomal recessive mutations in *IL2RB* (in M5) have been recently characterized in severe autoimmunity and viral susceptibility, reflecting the important functions of *IL2RB* in T and natural killer cells signalling and in maintaining immune tolerance [61]. Conceivably, the identification of these functional module-specific genes provides new insights for future studies aimed at understanding the molecular mechanisms underlying immunity, with potential applications in developing therapeutics for multisystem autoimmune diseases and cancer, particularly in understudied populations such as those examined in the current study. Furthermore, the current study reprioritized four genes with respect to their functional significance derived from common genetic variations in Jam (*UQCRFS1*), A-A (*PTPN6*), Jpn (*RALY*), and across Jam, A-A and Jpn (*ZMYM4*). Mutations in these genes cause mitochondrial disorders (manifesting in severe multi-systemic disorders [62]), inflammatory diseases [63], metabolic disease states [64], and energy dyshomeostasis [65], respectively. These findings provide a framework for closely studying the connections between common regulatory variants and rare protein-coding mutations in related health conditions.

The limitation of the current study relates primarily to its sample size. Despite that the study examined the largest cohorts of world-class sprint and power athletes assembled to date across West-African and East-Asian ancestral groups, enabling the detection of moderate to large genetic effects, it lacks the statistical power to identify genetic variants with small to modest effects (effect sizes ranging from 1.1 to 1.4). Another limitation is the absence of a close examination of the gene-environment interactions, particularly the role of epigenetic mechanisms, such as DNA methylation and miRNA regulatory networks, in driving elite sprint and power performance. Future largescale, prospective studies should incorporate detailed environmental exposure variables and systematically record athletes’ living and training conditions to thoroughly investigate the interplay between genetic variations and environmental factors, for uncovering the complex biology underlying elite sprint and power performance. Although the current study offers new insights into the molecular factors influencing physical performance in health and disease, well-designed functional studies in disease contexts are necessary to fully elucidate these findings before drawing further clinical implications.

## CONCLUSIONS

Taken together, we identified nine genes and their sentinel SNPs (where applicable) associated with world-class sprint and power performance in the first cross-ancestry GWAS of elite athletes. We elaborated on their functional roles and interactions within tissue- and cell-type-specific networks. Furthermore, we uncovered thirty-six novel genes involved in cellular immune responses and highlighted four genes linked to aging, neurological, blood and bone disorders in these elite cohorts. It is noteworthy that our work focused on underrepresented ancestral groups from West Africa and East Asia, comprising athletes of the highest performance calibre, thereby adding significant value to the current genomic landscape. Future studies are required to closely examine the impact of gene-environment interactions on elite athletic performance in large-scale, well-designed research. Overall, the current findings not only provide new biological insights into elite sprint and power performance but also lay the foundation for future research in understanding how elite sprint and power performance-related genes may influence health and disease, particularly in musculoskeletal, metabolic, immunological and neurological conditions.

## Data Availability

Access to all the elite athletic performance cohorts may be limited by participant consent and data sharing agreements; requests should be directed in the first instance *via* the corresponding authors. All other data are available in the main text or the supplementary materials available online at https://doi.org/10.6084/m9.figshare.26889823 (Supplementary Figures and Tables) and at https://doi.org/10.6084/m9.figshare.26890210 (Supplementary Data).

## Supplementary Materials

The supplementary materials are available online at https://doi.org/10.6084/m9.figshare.26889823 (Supplementary Figures and Tables) and at https://doi.org/10.6084/m9.figshare.26890210 (Supplementary Data).

## Consortium

### Sportgene Research Group

Emiliya S. Egorova^9^, Leysan J. Gabdrakhmanova^9^, Ekaterina A. Semenova^11,25^, Nikolay A. Kulemin^11^, Andrey K. Larin^11^, Rinat I. Sultanov^11^, Andrey V. Zhelankin^11^, Irina V. Haidukevich^14^, Irina L. Gilep^26^, Egor B. Akimov^27^
